# Cancer rates not explained by smoking: a county-level analysis

**DOI:** 10.1186/s12940-020-00613-x

**Published:** 2020-06-06

**Authors:** Douglas J. Myers, Polly Hoppin, Molly Jacobs, Richard Clapp, David Kriebel

**Affiliations:** 1 Department of Community and Environmental Health, College of Health Sciences, Boise State University, 1910 University Drive, Boise, ID 83725 USA; 2 Lowell Center for Sustainable Production, University of Massachusetts, Lowell, 1 University Avenue, Lowell, MA 01854 USA

**Keywords:** Cancer prevention, Smoking cessation, Epidemiology, Smoking-related cancers

## Abstract

**Background:**

Debates over the importance of “lifestyle” versus “environment” contributions to cancer have been going on for over 40 years. While it is clear that cigarette smoking is the most significant cancer risk factor, the contributions of occupational and environmental carcinogens in air, water and food remain controversial. In practice, most cancer prevention messaging focuses on reducing cigarette smoking and changing other personal behaviors with little mention of environmental chemicals, despite widespread exposure to many known carcinogens. To inform decision-making on cancer prevention priorities, we evaluated the potential impact of smoking cessation on cancer rates.

**Methods:**

Using cancer incidence data from 612 counties in the SEER database, and county-level smoking prevalences, we investigated the impact of smoking cessation on incidence for 12 smoking-related cancer types, 2006—2016. A multilevel mixed-effects regression model quantified the association between county-level smoking prevalence and cancer incidence, adjusting for age, gender and variability over time and among counties. We simulated complete smoking cessation and estimated the effects on county-level cancer rates.

**Results:**

Regression models showed the expected strong association between smoking prevalence and cancer incidence. Simulating complete smoking cessation, the incidence of the 12 smoking-related cancer types fell by 39.8% (54.9% for airways cancers; 28.9% for non-airways cancers). And, while the actual rates of smoking-related cancers from 2006 to 2016 declined (annual percent change (APC) = − 0.8, 95% CI = − 1.0 to − 0.5%), under the scenario of smoking elimination, the trend in cancer incidence at these sites was not declining (APC = − 0.1, 95% CI = − 0.4 to + 0.1%). Not all counties were predicted to benefit equally from smoking elimination, and cancer rates would fall less than 10% in some counties.

**Conclusions:**

Smoking prevention has produced dramatic reductions in cancer in the US for 12 major types. However, we estimate that eliminating smoking completely would not affect about 60% of cancer cases of the 12 smoking-related types, leaving no improvement in the incidence trend from 2006 to 2016. We conclude that cancer prevention strategies should focus not only on lifestyle changes but also the likely contributions of the full range of risk factors, including environmental/occupational carcinogens.

## Introduction

Cancer prevention efforts focus primarily on changing personal behaviors – including smoking, diet, exercise, sun safety, and adherence to screening. Of these, it is well-accepted that smoking is the most important, accounting for as much as 19% of all cancer incidence while other “lifestyle” factors have more modest contributions [1].

There is also a substantial body of evidence linking environmental chemicals to cancer, although how and whether this information should be used in cancer prevention initiatives is more controversial [2–4]. The U.S. President’s Cancer Panel (PCP), concluding their 2010 report on Reducing Environmental Cancer Risk stated: “*The burgeoning number and complexity of known or suspected environmental carcinogens compel us to act to protect public health, even though we may lack irrefutable proof of harm”* [2]. Some leading experts disagreed [3]. For example, writing in the New England Journal of Medicine, Willett, Colditz and Hiatt [5] argued that the PCP was misguided in its emphasis on environmental carcinogens: “*It is important not to detract from the fact that the major causes of cancer are smoking, overweight, and inactivity*.” But if talking about environmental carcinogens “detracts” from attention to smoking, overweight and inactivity, the public, policy-makers and clinicians might be forgiven for concluding that all people need to do to prevent cancer is to change their unhealthy behaviors.

Debates over “lifestyle” versus “environment” contributions to cancer are longstanding (going back at least as far as Doll and Peto’s 1980 report [6]) and unlikely to be resolved anytime soon because of inadequate human carcinogenicity data. We therefore proposed a different approach to provide perspective on this debate. With data relevant for county-level decision-making, we simulated the best possible case of a behavioral intervention to prevent cancer – the complete elimination of smoking – and then estimated the resulting reduction in the rate of cancer, focusing only on cancer sites for which smoking is a known risk factor. How much would cancer rates fall, and how much cancer would remain? Would this best-case scenario prevent the large majority of cancers? If not, perhaps additional modifiable risk factors should be sought and addressed.

Using a multi-level statistical model of county-level U.S. cancer incidence data, we investigated three questions: 1. What proportion of the cancer incidence at 12 smoking-related cancer sites would remain if smoking were completely eliminated? 2. How would the 11-year trend (2006–2016) in 12 smoking-related types of cancer have been different if smoking had been completely eliminated? 3. How would the county-by-county variability in cancer rates at the 12 smoking-related sites change if smoking were completely eliminated?

## Materials and methods

Cancer incidence data by county were obtained from 18 cancer registries participating in the Surveillance, Epidemiology, and End Results (SEER) program of the National Cancer Institute (NCI), representing the highest quality cancer U.S. registry data available and covering about 28% of the US population [7]. SEER data contain cancer incidence information, as well as patient demographics and clinical characteristics including stage and grade. Reliable individual smoking data are not available from SEER, so county-level prevalence data were used instead (see below). All counties in SEER were included in the analysis, except Alaska where the registry only includes Native Alaskans. Population data are also provided. Because all data were publicly available and not personally identifiable, Institutional Review Board approval was not required.

We chose to study 12 cancer types which are considered to be caused by smoking according to the U.S. Centers for Disease Control and Prevention [8]. These can be divided into two groups – three airways cancers and nine other smoking-related cancers.

### Airways cancers

1. Trachea, bronchus and lung (ICD-O-3 codes C33.9–34.9).

2. Larynx (C32.0–32.9).

3. Oral cavity and pharyngeal (C00–14.8).

### Other smoking-related cancers

4. Esophagus (C15.0–15.9).

5. Stomach (C16.0–16.9).

6. Colon and rectum (C18.0–20.9).

7. Liver (C22.0).

8. Pancreas (C25.0–25.9).

9. Kidney and renal pelvis (C64.9–65.9).

10. Urinary bladder (C67.0–67.9).

11. Cervix (C53.0–53.9).

12. Acute myeloid leukemia (ICD-O-3 histology codes 9840, 9861, 9865–9867, 9869, 9871–9874, 9895–9898, 9910–9911, and 9920).

As the SEER incidence data provide county of residence information, the county (*n* = 612) was the unit of analysis. Independent variables were available at either the individual level or county level. Individual level variables for each case were: year of diagnosis (2006–2016); gender; and age, in 5-year categories (20–24, …, 80–84). Cancer data for all races were included, but race effects were not modeled because of the very small numbers of non-whites in many of the SEER counties. At the county level we used the age-standardized calendar year- and gender-specific smoking prevalence, obtained from the Institute for Health Metrics and Evaluation [9]. These estimates were based on Behavioral Risk Factor Surveillance System (BRFSS) data, which were modeled to generate estimates of county level smoking prevalence for the entire US for the period between 1996 and 2012. The variable was defined as “prevalence of current daily cigarette smoking”.

Statistical analyses were performed using multilevel mixed-effects regression models in STATA/MP 16.0 [10]. We modeled cancer incidence rates by using observed cancer counts as the dependent variable and person-time at risk as the offset. The observed counts were modeled assuming a negative binomial distribution, based on the presence of over-dispersion; incidence rate ratios and 95% confidence intervals were generated. The fixed-effect part of the model included the following covariates (selected a priori and forced into the models): age, gender, calendar year, age-adjusted daily smoking prevalence by county and year. In models of the full 11-year period, a 10-year lag was used for the smoking data, which was the longest lag possible because the smoking data were available only from 1996 onwards. We also fit a model for the latest year, 2016, and in this case, we fit smoking data lagged 20 years. A random intercept was assumed by county and a random slope by year (different effects of calendar year by county). We also fit models stratified by gender rather than including gender as a covariate. After fitting the model, we used the “predict” command in STATA to generate predicted counts of cancers by county and year which were then converted to expected rates using county populations, which were then age-standardized to the gender-specific SEER population distribution for 2016. Smoking is a much stronger risk factor for airways cancers than the other nine sites, so to more accurately estimate the effects of smoking and its elimination, we fit models separately for these two subgroups.

Trends by year were computed from both the fixed- and random-effects parts of the model; likewise, the county-by-by county variation was also computed including the fixed- and random-effects parts of the model. We used the model to compare two scenarios: the actual incidence rates, assuming the observed lagged county smoking prevalences (lagged 10 years for the 11-year trend models; lagged 20 years for the 2016 only models), and the smoking eliminated scenario. To obtain these simulated results, the value of smoking prevalence in each county was set to zero. Predicted values were obtained under this scenario, again via STATA’s “predict” command, then age-standardized to the gender-specific SEER population distribution for 2016 as were values obtained using the actual smoking prevalence data. To evaluate the annual trends, we fitted a log-linear model to the rates predicted from the above regression models. The annual percent change (APC) was estimated as the 100 * exponential of (beta – 1) [11].

After fitting the model estimating the effects of smoking elimination (county smoking prevalence set to zero), we predicted the rates of the smoking-related cancers in each county in 2016. Using 2016 county populations as weights, we then evaluated the county-by-county distribution of cancer rates across the total SEER population (*n* = 64,999,165).

## Results

**Fig. 1:**
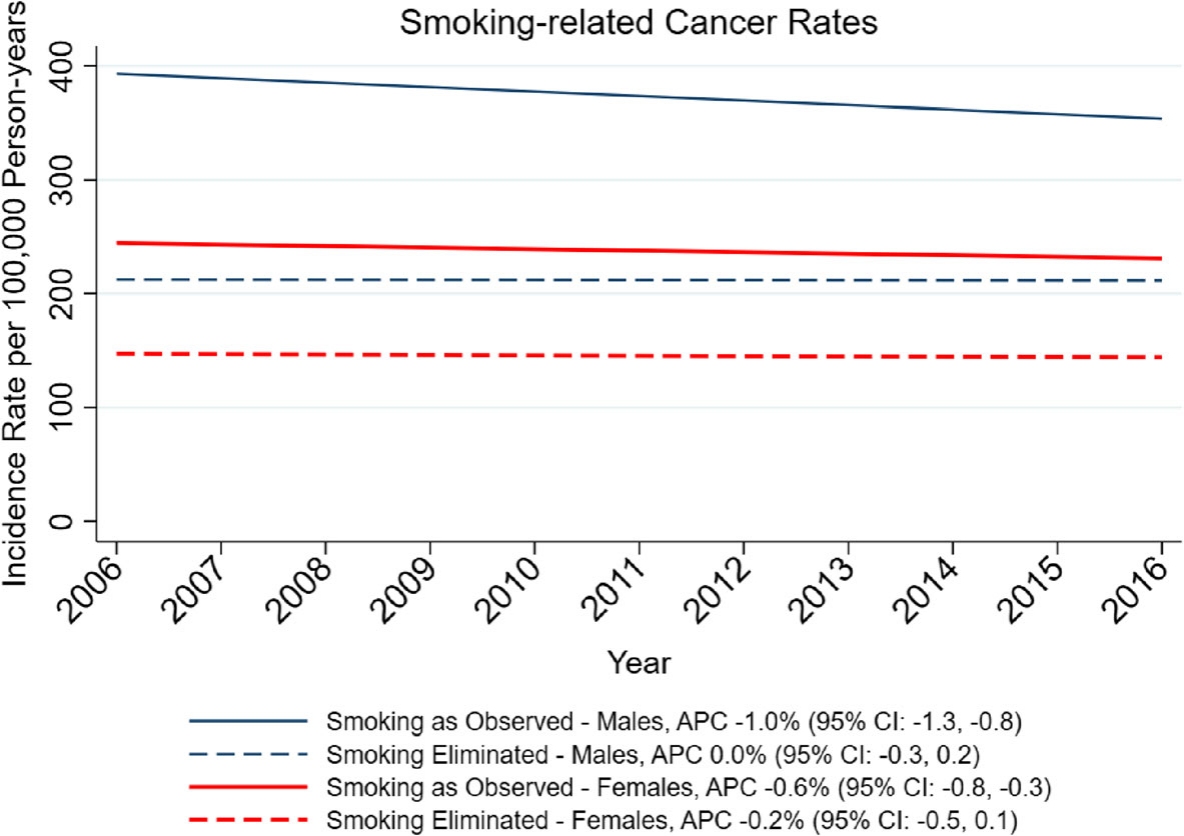
Gender-stratified trends of smoking-related cancers under two scenarios: as observed and the simulated elimination of smoking

The mean smoking prevalence for the 612 counties in 2016 (lagged 10 years) was 20.0% (SD = 5.0%, 5th percentile = 11.6%, 95th percentile = 28.2%). Cancer incidence rates were calculated by gender for each of the types of cancer associated with smoking, from the SEER data for the years 2006–2016 (Supplemental Table 1). These cancer sites included 35% of all cancers among women and 47% among men. For the 12 smoking-related sites combined, the annual age-standardized incidence rates declined over the 11 years in both men and women (Fig. 1, solid lines).

Next, three multivariate regression models, each accounting for the effects of age and gender and year of diagnosis, are shown in Table 1. The very strong increasing trend in incidence of cancer with age is easily seen in this table, as is the overall higher incidence among men compared to women – a more than 60% higher risk for the 12 smoking-related sites overall among men. One can also see the modest but clear decline in the incidence of these cancers over the time period 2006–2016; with 2006 set as the reference year, the relative risk of cancer declines over the years so that by 2016 the relative risk was 0.90, or a 10% decrease, compared to 2006. Fitting linear trends to the annual data for men and women, the annual percent changes were − 1.0% (95% CI = − 1.3% to − 0.8%) and − 0.6% (95% CI = − 0.8% to − 0.3%), respectively (combined: -0.8, 95% CI = − 1.0 to − 0.5%, Fig. 1).

**Table 1:** Incidence rate ratios for determinants (excluding smoking) of all cancers, smoking-related^a^ and non-smoking-related cancers^b^

	All Cancers		All Smoking-related Cancers		All Non-smoking-related Cancers	
Variable	*IRR*	*(95% CI)*	*IRR*	*(95% CI)*	*IRR*	*(95% CI)*
Age at diagnosis						
20–24	1	(Ref.)	1	(Ref.)	1	(Ref.)
25–29	1.64	(1.61–1.68)	2.09	(2.00–2.18)	1.56	(1.53–1.60)
30–34	2.56	(2.51–2.61)	4.03	(3.88–4.19)	2.30	(2.25–2.35)
35–39	3.86	(3.79–3.93)	6.80	(6.56–7.05)	3.31	(3.25–3.38)
40–44	6.12	(6.02–6.23)	11.98	(11.57–12.42)	5.01	(4.91–5.11)
45–49	9.85	(9.68–10.01)	21.76	(21.01–22.53)	7.55	(7.41–7.70)
50–54	15.81	(15.54–16.07)	39.80	(38.45–41.19)	11.34	(11.13–11.56)
55–59	23.44	(23.06–23.83)	59.41	(57.41–61.48)	16.84	(16.53–17.15)
60–64	33.25	(32.71–33.80)	87.89	(84.94–90.95)	23.60	(23.17–24.04)
65–69	46.43	(45.68–47.20)	130.40	(126.02–134.93)	31.83	(31.26–32.42)
70–74	55.36	(54.46–56.28)	171.15	(165.40–177.10)	35.93	(35.27–36.60)
75–79	62.19	(61.18–63.23)	208.92	(201.90–216.19)	38.17	(37.47–38.89)
80–84	65.10	(64.03–66.19)	232.89	(225.04–241.01)	38.07	(37.36–38.80)
Gender						
Women	1	(Ref.)	1	(Ref.)	1	(Ref.)
Men	1.16	(1.16–1.17)	1.61	(1.60–1.61)	0.96	(0.96–0.97)
Year of diagnosis						
2006	1	(Ref.)	1	(Ref.)	1	(Ref.)
2007	1.01	(1.00–1.03)	0.99	(0.97–1.02)	1.03	(1.01–1.04)
2008	1.01	(0.99–1.02)	0.99	(0.96–1.02)	1.01	(1.00–1.03)
2009	1.00	(0.98–1.02)	0.98	(0.95–1.00)	1.01	(0.99–1.03)
2010	0.98	(0.97–1.00)	0.95	(0.93–0.98)	1.00	(0.98–1.02)
2011	0.98	(0.97–1.00)	0.94	(0.92–0.97)	1.00	(0.99–1.01)
2012	0.97	(0.96–0.99)	0.95	(0.93–0.98)	0.98	(0.96–0.99)
2013	0.96	(0.94–0.97)	0.93	(0.91–0.96)	0.97	(0.95–0.98)
2014	0.97	(0.96–0.99)	0.95	(0.92–0.97)	0.97	(0.96–0.99)
2015	0.97	(0.96–0.99)	0.93	(0.91–0.96)	0.98	(0.97–1.00)
2016	0.95	(0.94–0.97)	0.90	(0.88–0.93)	0.97	(0.96–0.99)

Abbreviations: *CI* Confidence interval; *IRR* Incidence rate ratio; *Ref* Reference

^a^ Smoking-related cancer sites classified according to [8].

^b^ Estimates from three negative binomial regression models with random intercept on county and random slope on year. SEER 18 registries, 2006–2016, Alaska Native Tumor Registry excluded because of the lack of information on county

Table 2 shows three additional regression models, each accounting for the effects of age, gender, year of diagnosis as well as each county’s prevalence of current smokers. This table includes models stratified by airways and non-airways cancers (Table 2). For an initial descriptive model, the smoking prevalence data were categorized into six groups, with the lowest prevalence group (4.5 to 10% current smokers) as the reference. The strong association between smoking and these 12 types of cancer is clearly seen. In particular, the risk of airways cancers (second column of rate ratios in Table 2) increased strongly as the smoking prevalence increased. In the highest smoking counties (those with 22.9 to 38.3% current smokers), the risk of these cancers was more than double (RR = 2.19) that of the lowest smoking prevalence counties. Risk of the other smoking-related cancer sites also clearly increased as the smoking prevalence increased, but not as strongly, rising to RR = 1.32 – a 32% increase in risk in the highest smoking counties.

**Table 2:** Airways and non-airways stratified incidence rate ratios for determinants (including smoking) of smoking-related^a^ cancers^b^

	All smoking-related cancers		Smoking-related cancers of the airways		Other smoking-related cancers (non–airways)	
Variable	*IRR*	*(95% CI)*	*IRR*	*(95% CI)*	*IRR*	*(95% CI)*
Age at the diagnosis						
20–24	1	(Ref.)	1	(Ref.)	1	(Ref.)
25–29	2.09	(2.00–2.18)	1.69	(1.55–1.85)	2.21	(2.11–2.32)
30–34	4.03	(3.88–4.18)	2.99	(2.75–3.24)	4.35	(4.17–4.55)
35–39	6.80	(6.55–7.05)	5.65	(5.238–6.09)	7.19	(6.89–7.50)
40–44	11.98	(11.56–12.42)	12.60	(11.71–13.55)	11.86	(11.39–12.35)
45–49	21.76	(21.01–22.53)	30.23	(28.16–32.46)	19.18	(18.43–19.97)
50–54	39.79	(38.44–41.18)	62.32	(58.08–66.87)	32.88	(31.60–34.21)
55–59	59.36	(57.36–61.44)	105.06	(97.93–112.72)	45.37	(43.61–47.19)
60–64	87.73	(84.77–90.97)	162.40	(151.39–174.21)	65.05	(62.54–67.66)
65–69	130.08	(125.70–134.61)	248.44	(231.60–266.49)	94.28	(90.65–98.06)
70–74	170.58	(164.84–176.52)	331.66	(309.19–335.77)	122.6	(117.88–127.52)
75–79	208.16	(201.14–215.41)	392.41	(365.81–420.95)	154.49	(148.53–160.69)
80–84	232.11	(224.28–240.23)	405.43	(377.91–434.95)	182.83	(175.76–190.18)
Gender						
Women	1	(Ref.)	1	(Ref.)	1	(Ref.)
Men	1.46	(1.45–1.46)	1.35	(1.35–1.37)	1.53	(1.52–1.54)
Year of diagnosis						
2006	1	(Ref.)	1	(Ref.)	1	(Ref.)
2007	1.00	(0.98–1.02)	1.00	(0.97–1.03)	1.00	(0.99–1.02)
2008	1.00	(0.98–1.02)	1.00	(0.98–1.03)	1.01	(0.99–1.02)
2009	1.00	(0.98–1.01)	1.00	(0.98–1.03)	0.99	(0.97–1.01)
2010	0.99	(0.97–1.00)	0.99	(0.96–1.02)	0.98	(0.96–1.00)
2011	0.97	(0.95–0.99)	0.98	(0.95–1.00)	0.97	(0.95–0.98)
2012	0.99	(0.97–1.01)	0.99	(0.96–1.01)	0.99	(0.97–1.00)
2013	0.97	(0.95–0.99)	0.97	(0.94–0.99)	0.96	(0.95–0.98)
2014	0.99	(0.97–1.01)	0.99	(0.96–1.01)	0.99	(0.98–1.01)
2015	0.99	(0.97–1.01)	0.98	(0.96–1.01)	0.99	(0.97–1.01)
2016	0.97	(0.95–0.99)	0.97	(0.95–1.00)	0.97	(0.95–0.99)
Prevalence of daily smoking by county						
4.5–10.0%	1	(Ref.)	1	(Ref.)	1	(Ref.)
10.1–12.1%	1.05	(1.03–1.07)	1.09	(1.06–1.12)	1.05	(1.02–1.07)
12.2–15.6%	1.09	(1.07–1.11)	1.20	(1.17–1.23)	1.06	(1.04–1.08)
15.7–19.1%	1.17	(1.15–1.19)	1.36	(1.33–1.40)	1.11	(1.09–1.13)
19.2–22.8%	1.31	(1.28–1.34)	1.62	(1.58–1.67)	1.20	(1.17–1.22)
22.9–38.3%	1.58	(1.54–1.61)	2.19	(2.13–2.26)	1.32	(1.29–1.35)

Abbreviations: *CI* Confidence interval; *IR* Incidence rate

^a^ Smoking-related cancer sites classified according to [8]

^b^ Estimates from three negative binomial regression models with random intercept on county and random slope on year. SEER 18 registries, 2006–2016, Alaska Native Tumor Registry excluded because of the lack of information on county

The model summarized in Table 2 quantifies the effects on cancer incidence explained by age, gender, year and smoking. By fixing the values of any of these inputs to the model, it is possible to simulate different scenarios. To estimate the incidence rates of these 12 cancers over this time period in the SEER counties had smoking been completely eliminated, we again fit the model in Table 2, except that smoking prevalence was coded with a single continuous variable rather than six categories, and set to zero for all observations when obtaining predicted values.

As expected, under the scenario of complete smoking elimination, the incidence rates were substantially lower (Fig. 1, dashed lines) compared to the actual cancer incidence (Fig. 1, solid lines). For the final year, 2016, declines were 40.0 and 36.9% for males and females, respectively. Also, under the assumption that smoking had been completely eliminated, the modest downward trends in rates were no longer evident. The model predicted essentially no change in the cancer incidence over the 11-year period in either men or women.

**Fig. 2:**
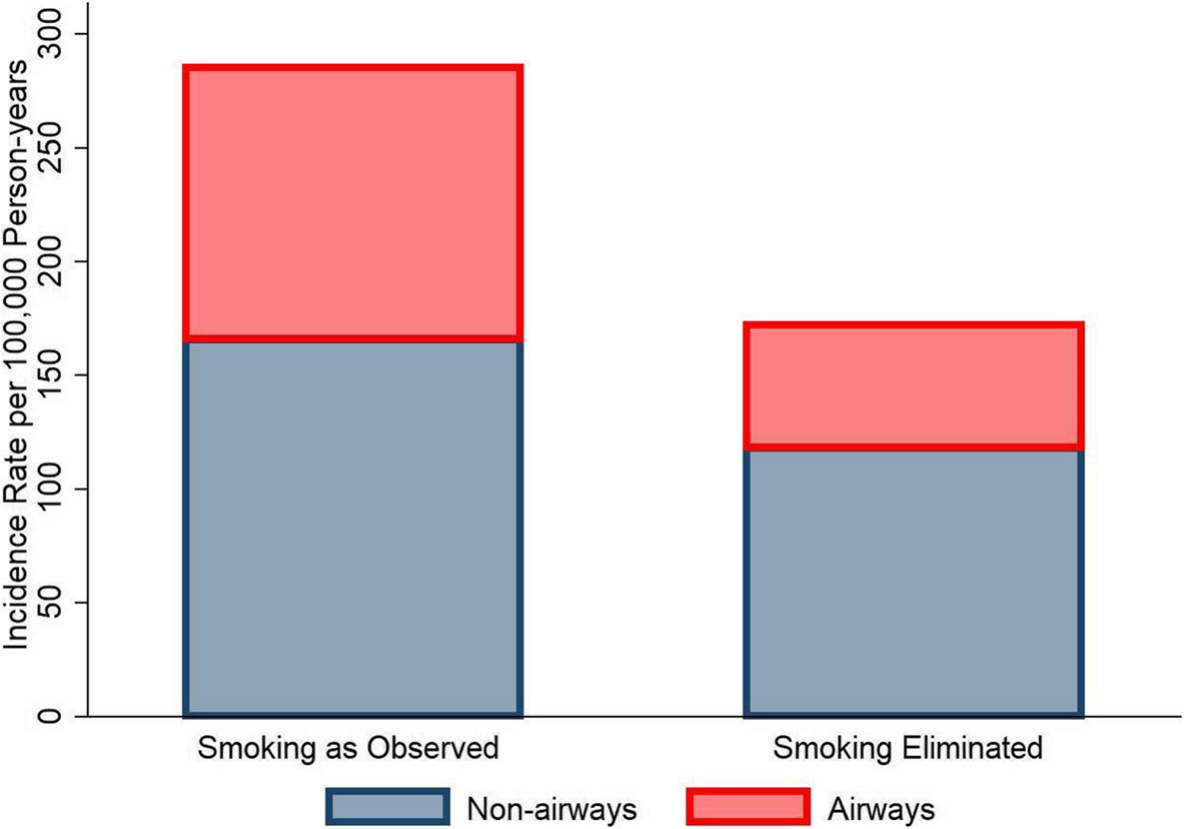
Smoking-related airways and non-airways cancer rates, 2016, under two scenarios: as observed and the simulated elimination of smoking

As noted, the impact of smoking is considerably stronger for the airways cancers than for the other smoking-related sites. Analyzing the effect of smoking elimination on these two groups separately in 2016 (with a 20-year lag for the smoking data), we found that incidence of the airways cancers would decline by 54.9%, while for the non-airways sites, the decline would be 28.9% (Fig. 2). Overall, there would be a reduction of 39.8% in the incidence of smoking-related cancers in 2016 if smoking were completely eliminated (using a 10-year lag would result in a 51.4% drop in cancers of the airways, a 23.4% drop of non-airway cancers and a combined decrease of 35.2%).

**Fig. 3:**
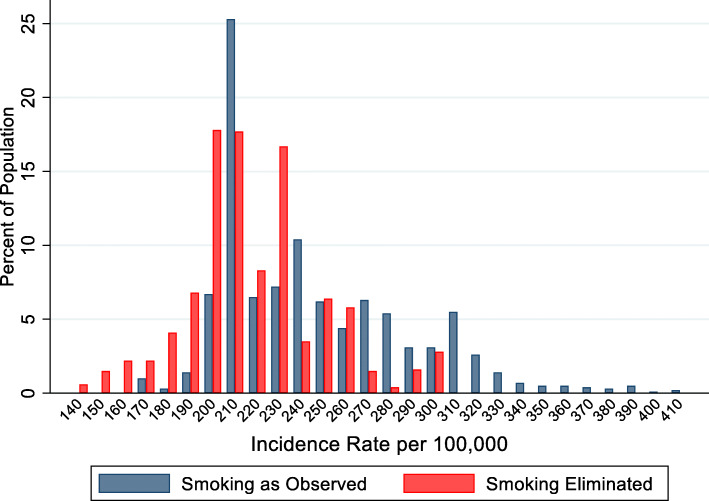
Population distribution of county-level smoking-related cancer rates, 2016, under two scenarios: as observed and the simulated elimination of smoking

We examined county-by-county variability under the smoking elimination scenario in 2016. Not all counties would see the same degree of cancer reduction from smoking elimination (Fig. 3). Not surprisingly, the counties that would see the largest benefits from smoking elimination have high smoking prevalences. For example, the 20 counties with the highest rates of smoking-related cancers in 2016 had an average smoking prevalence nearly twice the overall average (32% versus 18%) and all 20 counties were in Kentucky. But there would also be counties which would benefit less from smoking elimination. For example, there are five counties with predicted cancer rates after eliminating smoking that are greater than 280/100,000 (Fig. 3, right tail of the smoking eliminated distribution). These are all in the metropolitan areas of large cities: Jefferson County KY (Louisville) (smoking prevalence = 20.6%), Wayne and Macomb counties MI (Detroit) (smoking prevalence = 20.7 and 20.9%, respectively), Campbell County KY (Cincinnati) (smoking prevalence = 23.4%), and Jefferson Parish LA (New Orleans) (smoking prevalence = 23.8%). If smoking were eliminated, we predict that these counties would have smoking-related cancer rates that are about the same as the current average rate for all 612 SEER counties (left bar in Fig. 2). The model predicts that these five counties would see only an approximate 8% reduction in their rates of smoking-related cancers, far less than the overall average of about 40%, after total smoking elimination.

## Discussion

Overall cancer incidence in the U.S. has been falling over the past several decades [12]. This has been attributed largely to the reduction in smoking prevalence, especially among men. We were interested in investigating the role of changes in smoking habits on the incidence of cancer, and in particular we wanted to answer the hypothetical question: “Suppose smoking cessation were completely effective and smoking was entirely eliminated as a cause of cancer. How much cancer would still remain?” We focused therefore on cancer incidence at 12 sites known to be linked to smoking; cancer risk at other sites would not be affected by changes in smoking, and so we excluded them from our analyses.

We draw three main conclusions: First, by simulating the impact of the elimination of smoking on cancer rates, we estimated that about 60% of the cancers at tumor sites considered to be smoking-related would still occur (60% for males, 63% for females) in the absence of smoking. Second, under the same simulated condition of no smoking, the 11-year trend in the incidence rate of these 12 types of cancer would have been stable – neither rising nor falling – suggesting that smoking cessation is almost entirely responsible for the reductions in incidence that have occurred over this time period. Third, the degree to which smoking elimination would reduce cancer incidence rates would not be uniformly distributed across the country. We predict that some counties would have only modest reductions in cancer from eliminating smoking and by extension higher rates of cancers remaining. Future research might prioritize identifying additional factors explaining these elevated rates. Decision-makers should consider the potential contribution of other cancer risk factors in addition to smoking in designing comprehensive cancer prevention programs.

We compared our results to other estimates of tobacco’s contribution to cancer incidence. Islami and colleagues at the American Cancer Society recently published an extensive review of the potential for cancer prevention from addressing modifiable risk factors, including smoking [1]. Using an entirely different method, based on a synthesis of published estimates of the relative risks for different exposures, they concluded that in 2014, smoking was responsible for 19% of all incident cancers in the U.S. Parkin and colleagues [13] arrived at the same figure for the U.K. in 2010. Our estimate of 2016 data for the 40.9% of cancers that occur at the 12 smoking-related sites agrees very well with this: a reduction of 39.8% for 40.9% of all cancers translates to smoking being responsible for 16.3% of all cancers, not very different from their estimate of 19%. This gives us some confidence that our method, based on patterns in county-level data from the SEER registries, provided reasonable estimates for making national inferences.

Islami and colleagues also estimated the proportions of cancers in 2014 attributable to other lifestyle factors [1]. After smoking, the most important were excess body weight (7.8%), alcohol (5.6%) and physical inactivity (2.9%). These relatively minor contributions support our choice to focus this paper on smoking as the single most important behavioral risk factor for cancer.

The role of tobacco control in the decline in cancer rates has been widely recognized [14]. Thun and colleagues wrote: “*The most striking success in [cancer] primary prevention is undoubtedly tobacco, where falling consumption has resulted in marked reductions in the incidence and death rates from … tobacco-related cancers among men …*” [15]. Our analyses did not include cancers at the other sites, un-related to smoking, but Han and colleagues did [16]. Over a longer time period, 1975–2004, they found that for cancers unrelated to tobacco (or cancer screening which complicates the picture for several sites such as prostate and breast), cancer incidence *increased* in both men (0.88% annually for whites, 0.12% annually for blacks) and women (0.69% annually for whites, 0.52% annually for blacks).

Without detracting from the importance of tobacco control, these findings suggest that progress in the primary prevention of cancer must include many strategies, not only those focused on healthy lifestyles but also reduction of carcinogen exposures wherever possible [17, 18]. Such exposures include urban air pollution, particularly diesel exhaust, water pollution, pesticides in food, ionizing radiation and work-related chemicals [2]. Using the same SEER county cancer incidence data (2006–2010), Jagai and colleagues found a strong association between all-site cancer incidence and a measure of overall environmental quality, the Environmental Quality Index (which, developed by the U.S. EPA, combines measures of environmental quality in five domains: air, water, land, built environment and sociodemographic factors) [19].

As noted in the introduction, there are disagreements in the cancer prevention community about the importance of addressing environmental exposures [3]. Some argue that because the proportion of cancers caused by environmental exposures (often called the attributable fraction) is small relative to smoking and other health behaviors, control of the former does not warrant attention as a prevention strategy. There are several problems with this argument. First, cancers are known to have multiple causes and the very concept of apportioning different fractions of a disease rate to different single causes is misleading [20]. For a multifactorial disease, attributable fractions must add to more than 100% — probably much more, but calculations of attributable fractions for cancer causes often do not [1, 21].

A second major problem with the calculation of attributable fractions is that they measure the impact of the complete removal of the exposure. The complete elimination of smoking is probably impossible, at least in a democratic society. The most recent U.S. Preventive Services Task Force evaluation of the effectiveness of smoking cessation strategies found the best strategies have success rates of 28% or less [22]. Therefore, if we want to decide how much to prioritize tobacco control strategies, we should understand that 19% – Islami’s estimate of the percent of all cancers caused by smoking – is probably not a realistic goal for prevention. In our analyses, we chose to use the unrealistic assumption of smoking elimination because our intent was to place a lower bound on the proportion of the smoking-related cancers that would *not* be prevented by tobacco control. Also, we wanted to be able to compare our results to others, like Islami, who used a more conventional approach to estimating attributable fractions.

An important limitation of this study was the lack of individual level smoking data. Using lagged county-level smoking prevalence was an imprecise proxy for personal smoking data with individual information on amount smoked, duration, etc. Data availability prevented us from applying a 20-year lag to the model for the full 11-year period (Fig. 1), but the results for 2016 using the 10- versus 20-year lagged smoking data were very similar (35.2% versus 39.8%, respectively). We therefore doubt that the time trend in Fig. 1 would have been importantly changed if the longer lag could have been used. Despite the limitations in the smoking data, we were reassured that our estimate of the size of the reduction in cancer incidence assuming smoking elimination was in good agreement with others who have used entirely different methods to arrive at a similar result [1]. Another limitation is that smoking, whether measured individually or geographically, is often correlated with other cancer risk factors including air pollution, occupational exposures and dietary factors. We were unable to remove the potentially confounding or modifying effects of these factors on estimates of the effect of smoking elimination. This would likely lead to an overestimation of the reduction in cancers observed by simulating the elimination of smoking. A strength of the ecological assessment of smoking patterns is that it should capture variability in secondhand smoking as well as active smoking. Therefore, these estimates of the benefits of smoking cessation would also include cancer prevention from eliminating secondhand exposure.

## Conclusions

While smoking cessation unquestionably continues to be a very important cancer prevention strategy, it cannot be anticipated to prevent the majority of cancers, even those for which smoking is a known risk factor. Particularly in counties where incidence rates of cancer would remain high even if smoking cessation programs were 100% effective, focusing only on smoking cessation may give the public and government leaders the false impression that other prevention strategies are unimportant. In these counties in particular, cancer prevention strategies should include policies and programs to reduce other known cancer risk factors, including environmental and occupational exposures, at the same time that healthy lifestyles continue to be promoted.

## Supplementary information

**Additional file 1.**

## Data Availability

Dataset analyzed during the current study is publicly available at the Surveillance, Epidemiology and End Results Program (SEER) repository, https://seer.cancer.gov/data/.

## Abbreviations

PCP: U.S. President’s cancer panel
SEER: Surveillance, epidemiology, and end results
NCI: National cancer institute
