# Supplementary material for: Cancer rates not explained by smoking: a county-level analysis
Source: Environ Health. 2020 Jun 6;19:64. doi: 10.1186/s12940-020-00613-x (PMC7276087; doi:10.1186/s12940-020-00613-x)
Supplement: Supplementary file 1 — Additional file 1. [file 12940_2020_613_MOESM1_ESM.docx]

| **Supplementary Table 1. Incidence rates of smoking-related cancer by site^a^. SEER 18 registries, 2006-2016^b^.** | | | | | | | | |
| --- | --- | --- | --- | --- | --- | --- | --- | --- |
|  | Females | | | | Males | | | |
|  |  |  | Age-standardized incidence^c^ | |  |  | Age-standardized incidence^c^ | |
| Site | Cases | Crude IR | IR | (95% CI) | Cases | Crude IR | IR | (95% CI) |
| All cancers | 2,001,698 | 579.7 | 603.7 | (602.9-604.5) | 2,128,581 | 640.5 | 689.5 | (688.6-690.4) |
|  |  |  |  |  |  |  |  |  |
| All smoking-related sites | 694,212 | 201.0 | 211.5 | (211.0-211.9) | 994,932 | 299.4 | 320.5 | (319.9-321.1) |
|  |  |  |  |  |  |  |  |  |
| Airways smoking-related | 278,789 | 80.7 | 85.9 | (85.6-86.2) | 374,081 | 112.6 | 121.24 | (120.9-121.6) |
| Trachea, bronchus and lung | 239,740 | 69.4 | 74.1 | (73.8-74.4) | 270,035 | 81.3 | 88.3 | (88.0-88.6) |
| Larynx | 6,046 | 1.8 | 1.8 | (1.8-1.9) | 24,375 | 7.3 | 7.9 | (7.8-8.0) |
| Oral cavity and pharyngeal | 33,003 | 9.6 | 10.0 | (9.9-10.1) | 79,671 | 24.0 | 25.1 | (24.9-25.3) |
| Non-airways smoking-related | 397,420 | 115.1 | 120.1 | (119.7-120.4) | 564,751 | 170.0 | 181.5 | (180.0-181.9) |
| Esophagus | 8,213 | 2.4 | 2.5 | (2.5-2.6) | 31,469 | 9.5 | 10.2 | (10.1-10.3) |
| Stomach | 27,152 | 7.9 | 8.2 | (8.1-8.3) | 43,310 | 13.0 | 13.9 | (13.8-14.0) |
| Colon and rectum | 169,860 | 49.2 | 51.4 | (51.2-51.7) | 197,739 | 59.5 | 63.3 | (63.1-63.6) |
| Liver | 18,003 | 5.2 | 5.5 | (5.4-5.6) | 56,100 | 16.9 | 17.8 | (17.7-17.9) |
| Pancreas | 51,205 | 14.8 | 15.6 | (15.5-15.7) | 57,234 | 17.2 | 18.4 | (18.3-18.6) |
| Kidney and renal pelvis | 51,978 | 15.0 | 15.8 | (15.6-15.9) | 91,540 | 27.6 | 29.1 | (28.9-29.2) |
| Urinary bladder | 38,727 | 11.2 | 11.9 | (11.8-12.0) | 127,396 | 38.3 | 41.5 | (41.2-42.7) |
| Cervix | 36,804 | 10.7 | 10.6 | (10.5, 10.8) | -- | -- | -- | -- |
| Acute myeloid leukemia | 13,481 | 3.9 | 4.1 | (4.0-4.1) | 16,063 | 4.8 | 5.1 | (5.0-5.2) |

Abbreviations: CI, confidence interval; IR, incidence rate.

^a^ Smoking-related cancer sites classified according to.(8)

^b^ Alaska Native Tumor Registry excluded because of the lack of information on county.

^c^ Rates standardized on the age distribution of the population covered by SEER registries in 2016.
